# The interaction between ros generation and fas signaling in regulation of lps-induced lung epithelial cell apoptosis

**DOI:** 10.1186/2197-425X-3-S1-A435

**Published:** 2015-10-01

**Authors:** W-C Lin, C-W Chen, C-F Lin, C-P Chang

**Affiliations:** Department of Internal Medicine, National Cheng Kung University Medical College and Hospital, Tainan, Taiwan Province of China; National Cheng Kung University Medical College and Hospital, Tainan, Taiwan Province of China; Taipei Medical University, Taipei, Taiwan Province of China; National Cheng Kung University Medical College, Tainan, Taiwan Province of China

## Introduction

Accumulating evidence indicates that oxidative stress contributes to the pathogenesis of acute lung injury (ALI). Previous studies have demonstrated that LPS-induced reactive oxygen species (ROS) mediates lung epithelial cell apoptosis. Fas/FasL signaling is an important cellular pathway in the induction of lung epithelial cell apoptosis in ALI.

## Objectives

To determine the interaction between ROS generation and Fas activation in regulation of LPS-induced lung epithelial A549 cell apoptosis.

## Methods

Intracellular ROS was measured using 2',7'-dichlorofluorescein diacetate as fluorescent probes by flow cytometry in LPS-treated A549 cells. Addition of superoxide dismutase (SOD, O_2_^·-^ scavenger), catalase (H_2_O_2_ scavenger), or sodium formate (OH^-^ scavenger) was performed to determine the specificity of ROS detection. To determine the source of ROS generation induced by LPS, diphenylene iodonium (DPI), a known inhibitor of NADPH oxidase, or rotenone, an inhibitor of mitochondrial electron transport chain, were pretreated, and their effects on ROS generation was examined. LPS-induced cell apoptosis was analyzed by Annexin-V staining followed by flow cytometry in the presence or absence of ROS scavengers. Furthermore, Fas blocking antibody ZB4 and Fas siRNA were pretreated to verify the role of Fas signaling in LPS-induced ROS generation and cell apoptosis. To determine the effects of ROS on Fas signaling, Fas levels of A549 in response to LPS were measured by flow cytometry in the presence or absence of ROS scavengers. The downstream caspase-3 and -8 activity were examined, and their inhibitors were treated to confirm the effects of caspase-3 and -8 on LPS-induced apoptosis.

## Results

We have demonstrated that pretreatment of ROS scavengers (SOD, catalase and sodium formate) diminished LPS-induced intracellular ROS generation as well as inhibited A549 cell apoptosis. Similarly, the DPI and rotenone also prevented from LPS-induced apoptosis through inhibition of ROS generation. Furthermore, we found that inhibiting ROS generation attenuated LPS-induced Fas up-regulation and inhibited the activation of caspase-8 and -3. Addition of caspase-8 inhibitor Z-IETD-FMK or caspase-3 inhibitor Z-DQMD-FMK diminished LPS-induced cell apoptosis. Of note, blocking Fas by Fas antibody ZB4 or siRNA inhibited LPS-induced cell apoptosis, but was not associated with ROS production.

## Conclusions

Our data show that LPS induces superoxide, hydrogen peroxide and hydroxyl radical production either from NADPH oxidase or from mitochondrial electron transport chain in A549 cells. The LPS-induced ROS generation results in A549 apoptosis through Fas up-regulation and downstream caspase-8 and -3 activation. Blocking Fas signaling has no effect on regulation of LPS-induced ROS generation while inhibiting cell apoptosis.

## Grant Acknowledgment

This work was supported by the Ministry of Science and Technology, Taiwan (MOST 103-2314-B-006 -063).

